# Inflammatory Markers Stratify Surgical Outcomes in Pediatric Airway Reconstruction

**DOI:** 10.1002/lary.70241

**Published:** 2025-11-27

**Authors:** Nomongo Dorjsuren, Hrithik Praveen, Kalpnaben Patel, Shilin Zhao, Alexander Gelbard, Christopher Wootten

**Affiliations:** ^1^ Vanderbilt University School of Medicine Nashville Tennessee USA; ^2^ Surgical Outcomes Center for Kids (SOCKs) Vanderbilt University Medical Center Nashville Tennessee USA; ^3^ Department of Otolaryngology‐Head and Neck Surgery Monroe Carell Junior Children's Hospital at Vanderbilt University Medical Center Nashville Tennessee USA

**Keywords:** biomarkers, erythrocyte indices, humans, laryngostenosis, pediatrics, retrospective studies, surgical airway reconstruction, tracheal stenosis, treatment outcome, triple‐endoscopy

## Abstract

**Objectives:**

Red blood cell distribution width (RDW) predicts surgical success in adult patients undergoing open airway reconstruction for laryngotracheal stenosis, but similar biomarkers in pediatrics remain unidentified. This study identifies predictors of outcomes in pediatric patients undergoing triple endoscopy or surgical airway reconstruction.

**Methods:**

A retrospective cohort study of 191 pediatric patients who underwent triple endoscopy or surgical airway reconstruction at an aerodigestive center between 4/18/2013 and 4/17/2023 was completed. Ninety‐eight patients with lab values within 2 months of the procedure were included. Main outcome measures were prosthesis‐free breathing at last follow‐up, hospitalization length, and follow‐up duration.

**Results:**

Lower RDW values were associated with prosthesis‐free breathing at last follow‐up (*p* = 0.042) and shorter hospitalizations (*p* < 0.001). Higher monocyte‐to‐lymphocyte ratio (MLR) and systemic inflammation response indexes (SIRI) correlated with increased length of hospitalization (*p* = 0.003 and *p* = 0.01). Neutrophil‐to‐lymphocyte ratio (NLR), platelet‐to‐lymphocyte ratio (PLR), and tracheomalacia were associated with longer follow‐up periods (*p* = 0.04). Patients with tracheomalacia and OSA prior to intervention were more likely to require tracheostomy at last follow‐up (*p* = 0.005 and *p* = 0.041).

**Conclusions:**

RDW may predict long‐term surgical success and outcomes in pediatric patients with complex airway, pulmonary, and upper digestive tract disorders. Serologic markers including SIRI, MLR, NLR, and PLR may also predict outcomes. Patient demographics and surgical type did not correlate with long‐term outcomes, but patients with tracheomalacia and OSA were more likely to require tracheostomy at later time points.

**Level of Evidence:**

3.

## 1 Introduction

Advances in critical care have increased the population of patients with complex respiratory and gastrointestinal symptoms that require intensive interdisciplinary management [1, 2]. These disorders, known collectively as aerodigestive conditions, include diagnoses such as laryngotracheal stenosis, laryngotracheoesophageal clefts, airway malacia, and other congenital airway or esophageal anomalies, gastroesophageal reflux, eosinophilic esophagitis, and oropharyngeal dysphagia with aspiration. Early‐in‐life intubation in the intensive care unit may lead to airway pathology secondary to complex interactions between host immunity and antigens in the injured airway. Lasting complications such as tracheal stenosis, vocal cord paralysis, tracheomalacia, and subglottic stenosis affect 0.63%–2% of neonates [3, 4]. Similarly, other inflammatory conditions driving aerodigestive disease that arise later in childhood have been related to NICU admissions [5, 6, 7]. Management of these aerodigestive conditions requires a multidisciplinary approach that includes a comprehensive endoscopic investigation—the “triple scope,” which includes rigid microlaryngoscopy and bronchoscopy, flexible bronchoscopy, and esophagogastroduodenoscopy. Interventions follow, with more severe cases requiring airway reconstruction.

Significant variability in surgical outcomes exists among children with aerodigestive disorders undergoing airway reconstructive surgery. Prior studies have shown that operation success rates, defined as decannulation rate if tracheostomy is present, may vary between less than 50%–95% depending on comorbidities or surgical approach [8, 9]. For this reason, a method of stratifying aerodigestive pediatric patients is needed to understand which patients are physiologically appropriate for major laryngotracheal surgery. Serological measurements, such as red blood cell distribution width (RDW), neutrophil‐to‐lymphocyte ratio (NLR), monocyte‐to‐lymphocyte ratio (MLR), and platelet‐to‐lymphocyte ratio (PLR), represent a growing area of interest for use as clinical biomarkers for a variety of inflammatory conditions [10, 11, 12].

This study aims to evaluate whether serological inflammatory markers and clinical risk factors can serve as predictors of surgical outcomes in pediatric aerodigestive patients undergoing airway reconstruction. We hypothesized that these biomarkers could help stratify patients by disease severity and predict their likelihood of successful surgical outcomes. To assess this, our primary outcome was prosthesis‐free breathing at last follow‐up—a direct measure of surgical success. Secondary outcomes included the need for additional airway procedures, length of hospitalization, and duration of follow‐up, which reflect the broader clinical burden of disease. By linking inflammatory markers to these outcomes, we sought to determine whether such markers could aid in identifying which children are optimal candidates for airway reconstruction and which may require alternative management strategies.

## 2 Methods

This research project was approved by the Vanderbilt University Medical Center Institutional Review Board (IRB 180555), which waived the requirement for informed consent. An initial 191 pediatric patients who underwent a triple endoscopy or surgical airway reconstruction between the dates of 18 April 2013 and 17 April 2023, at Vanderbilt's Complex Aerodigestive Evaluation Team (CADET) were screened. Patients were identified by using Current Procedural Terminology codes including 20,910, 31,570, 31,551, 31,582, and 31,587. Of these, 98 patients were included based on the presence of serological labs that were collected on the day of their procedure prior to the induction of anesthesia, or up to 2 months before.

Patient characteristics were extracted from the medical record which included demographic information, serological lab values, medical history, comorbidities, operation type, complications, and whether patients had prosthesis‐free breathing at their last follow‐up visit. McCaffrey grade [13, 14, 15] was also collected, which classifies LTS based on the length of stenosis and the subsites involved. The RDW value collected was RDW‐CV expressed in a percentage. All further references to RDW in this manuscript refer to RDW‐CV. Patient groups were categorized based on the cause of airway stenosis: iatrogenic, congenital, or traumatic. Stenosis was classified as iatrogenic if it developed within a year of prolonged intubation, which was most commonly due to premature birth. Congenital causes of stenosis included those due to congenital syndromes or otherwise unexplained airway malformations.

Serological values were collected from patient CBC and BMPs. Serological ratios were calculated using raw counts of lymphocytes, monocytes, neutrophils, and platelets. These ratios included neutrophil‐to‐lymphocyte ratio (NLR), monocyte‐to‐lymphocyte ratio (MLR), platelet‐to‐lymphocyte ratio (PLR), and systemic inflammation response index (SIRI) which is calculated by multiplying neutrophil count by monocyte count, then dividing by lymphocyte count.

Operation types were categorized into triple endoscopy alone, triple endoscopy with endoscopic airway intervention (balloon dilation or steroid injection), and surgical airway reconstruction. Surgical reconstructions were further subclassified into tracheal resection, cricotracheal resection, extended cricotracheal resection with placement of cartilage graft, tracheoplasty, slide tracheoplasty, supraglottoplasty, and laryngotracheal reconstruction with cartilage graft augmentation. Laryngotracheal reconstruction was subdivided based upon costal cartilage graft (CCG) site, which could either be isolated posterior CCG, isolated anterior CCG, both anterior and posterior CCG, and endoscope posterior CCG.

### 2.1 Statistical Analysis

Univariate analysis was completed to determine the impact of demographic, operation subtype, and serological values on primary and secondary outcomes. These tests included analysis of variance, the Wilcoxon test, Spearman correlation, Pearson's *χ* ^2^ test, and Fisher's exact test as appropriate. To identify independent risk factors for prosthesis‐free breathing, multivariate analysis and multivariate analysis of variance were conducted. Variables that met the inclusion threshold of *p* < 0.20 in univariate analysis were incorporated into the multivariate model. Results were expressed as effect sizes with corresponding confidence intervals, with statistical significance defined at *p* < 0.05.

## 3 Results

### 3.1 Patients and Disease Characteristics

Of the 191 patients screened, 98 patients met the inclusion criteria of having serological lab values within 2 months leading up to their procedure. Of the 98 patients, 42 had serological data collected on the day of their procedure, prior to anesthesia induction. The median time between blood draw and procedure was 2 days (IQR 0–27 days). Ninety‐five of these patients had CBC values available, of which 73 had CBC with differential that allowed for further calculation of NLR, PLR, MLR, and SIRI. The median (IQR) age was 22 (5–81) months. 64 (65%) were male; 60 (61%) were white, 29 (30%) were black or African American, 5 (5%) were multiracial, 2 (2%) were Asian, and 2 (2%) were Hispanic.

Iatrogenic causes were the most common cause of airway stenosis, impacting 67 of the 98 patients. Although prolonged intubation was the cause of iatrogenic injury as outlined in the methods, one exception was a patient who developed airway difficulties following cardiothoracic surgery. Congenital causes of stenosis affected 30 patients. Two patients had a traumatic cause of airway stenosis, both of which were following burn inhalation injury. One patient was included as both iatrogenic and congenital, due to airway stenosis existing since birth which later worsened to include tracheomalacia following intubation.

### 3.2 Characterization of Laryngotracheal Stenosis and Its Treatments

Thirty‐six of the 98 patients had recorded McCaffrey grades, but among them, those with an iatrogenic cause of stenosis were more likely to have higher grades. A total of 54.5% of patients with recorded McCaffrey grades with an iatrogenic cause of stenosis had a McCaffrey grade of 2 or greater. Patients with congenital causes of LTS were younger at the time of intervention median (IQR) was 5.5 (2–13) months. On the other hand, patients with traumatic causes had higher median (IQR) ages of 110 (98–122) months (Table 1).

**TABLE 1:** Patient characteristics and preoperative characteristics.

Characteristic	Total (*N* = 98)	Iatrogenic (*N* = 67)	Congenital (*N* = 30)	Traumatic (*N* = 2)
Age at Surgery in months, median (IQR)	22 (5–81)	43 (7.5–98)	5.5 (2–13)	110 (98–122)
Male sex, No. (%)	64 (65%)	43 (64%)	21 (70%)	1 (50%)
Insurance				
Private	34 (35%)	23 (33%)	10 (33%)	1 (50%)
Public	51 (52%)	35 (52%)	17 (57%)	0 (0%)
Military	8 (8%)	5 (7%)	3 (10%)	0 (0%)
None	5 (5%)	4 (6%)	0 (0%)	1 (50%)
McCaffrey Grade				
Grade 1	17 (47%)	11 (41%)	7 (77%)	0 (0%)
Grade 2	6 (19%)	5 (18.5%)	1 (11%)	0 (0%)
Grade 3	10 (27%)	9 (33%)	1 (11%)	0 (0%)
Grade 4	3 (9%)	3 (11%)	0 (0%)	1 (50%)
Race/ethnicity				
White	56 (57%)	39 (68%)	20 (67%)	2 (100%)
Black	29 (30%)	24 (36%)	5 (17%)	0 (0%)
Asian	2 (2%)	1 (1%)	1 (3%)	0 (0%)
Hispanic	5 (5%)	3 (4%)	2 (6.7%)	0 (0%)
Other	6 (6%)	0 (0%)	2 (6.7%)	0 (0%)
Smoking exposure	16 (18%)	15 (26%)	1 (3%)	0 (0%)
RDW‐CV in %, median (IQR)	14 (12.9–16.45)	13.6 (12.7–16.1)	14.5 (13.7–16.4)	15 (14.1–15.8)
Follow up in days, median (IQR)	542.5 (187–1065)	527 (208–1046)	624 (207–1090)	180 (173–187)

*Note*: The McCaffrey grade classifies laryngotracheal stenosis based on subsites involved and the length of stenosis.

Abbreviation: IQR, interquartile range.

Laryngotracheal reconstruction (LTR) was the most common surgical intervention overall, with 58.1% of patients undergoing surgery receiving it. Specifically, LTR with both anterior and posterior grafts was received by 41.8% of patients, followed by tracheoplasty (14.0%), then tracheal resection (11.6%) and LTR with isolated posterior CCG (11.6%) (Table 2). Two patients had complications from their operations, due to subcutaneous air requiring emergency intubation and infection requiring tracheostomy. There were no significant differences between length of hospitalization or length of follow‐up and surgical intervention type. The type of surgical intervention did not significantly correlate with the primary outcome of prosthesis‐free breathing. Eighty patients (82%) had prosthesis‐free breathing at their last follow‐up.

**TABLE 2:** Operative characteristics.

Characteristic	Patients, No. (%) (*N* = 98)	Length of hospitalization, d mean (SD)	Length of follow‐up, y mean (SD)	Additional procedures Average ± SD^a^
Triple endoscopy	**62 (76%)**	**28 (16)**	**1.4 (2.4)**	**1.63 (2.66)**
Balloon dilation	18 (23%)	19 (58)	0.6 (1.7)	1.22 (1.86)
Steroid injection	27 (34%)	29 (81)	1.3 (2.5)	1.24 (2.93)
Surgical reconstruction	**43 (44%)**	**24 (29.5)**	**1.9 (1.6)**	**2.28 (2.43)**
Tracheal resection	5	15 (22)	1.2 (1.3)	1.8 (1.79)
Cricotracheal resection	4	42 (47)	2.0 (2.3)	1.5 (1)
ECRCG	2	12 (2)	1.8 (1.8)	6.5 (2.12)
Tracheoplasty	6	44.5 (65)	1.2 (0.9)	1.17 (2.04)
Slide tracheoplasty	1	40 (40)	0.1 (0.1)	2 (NA)
Supraglottoplasty	4	5 (5)	3.0 (2.6)	0.5 (0.58)
LTR	**25**	**25 (34)**	**1.8 (1.9)**	**2.52 (2.5)**
Isolated posterior CCG	5	47 (32)	2.3 (1.9)	0 (NA)
Endoscope posterior CCG	1	18 (18)	0.5 (0.5)	5 (NA)
Isolated anterior CCG	1	8 (8)	0.5 (0.5)	1.6 (1.34)
Both anterior and posterior graft	18	23.5 (15)	1.1 (2.3)	3.11 (2.7)

*Note*: Bold values indicates statistically significant *p* values.

Abbreviations: CCG, costal cartilage graft; ECRCG, extended cricotracheal resection with cartilage graft; LTR, laryngotracheal reconstruction.

^a^ Additional number of procedures was counted for the next year after initial intervention.

### 3.3 Univariate Analysis of Inflammatory Serologies

**FIGURE 1:**
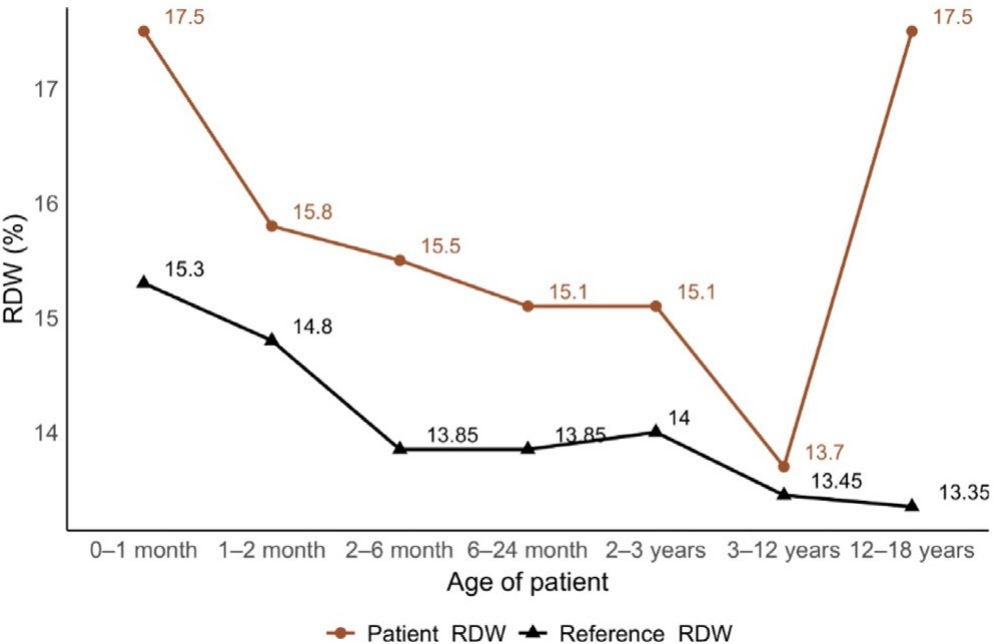
Comparison of patient RDW values to pediatric reference ranges. [Color figure can be viewed in the online issue, which is available at www.laryngoscope.com]

Median (IQR) RDW value was 14.0% (12.9%, 16.45%). Patients with traumatic LTS had the highest median RDW 15.0% (Table 1). Patients with congenital causes of LTS had the longest median (IQR) follow‐up period at 624 (207–1090) days whereas patients with traumatic causes of LTS had the shortest follow‐up durations (180 days). RDW values were compared to serological reference ranges at every age group (Figure 1) and were elevated in our patient cohort (*p* < 0.05). Lower RDW values were associated with shorter hospitalizations (*p* < 0.001).

When looking at other serological ratios, higher MLR and SIRI correlated with increased length of hospitalization (*p* = 0.003 and *p* = 0.01, respectively). However, they were not significantly related to our primary outcome measure of prosthesis‐free breathing. Increased NLR and PLR were associated with decreased length of follow‐up. However, nine patients with increased inflammatory serologies passed away within a few years of operation, which may explain prematurely reduced follow‐up durations (Table 3).

**TABLE 3:** Serological values and primary outcomes.

Characteristic	Prosthesis‐free breathing last follow‐up median	Breathing with prosthesis last follow‐up median	Length of hospitalization, y correlation	Length of follow‐up, y correlation
RDW‐CV in %	**13.85**	**15.1**	**0.37**	−0.21
MLR	0.26	0.31	**0.34**	−0.18
SIRI	1.17	1.86	**0.3**	−0.18
NLR	1.594	1.638	0.11	**−0.25**
PLR	103.1	106.4	−0.1	**−0.25**

*Note*: Bold values indicates statistically significant *p* values. Spearman correlation.

### 3.4 Multivariate Analysis of Inflammatory Serologies vs. Comorbidities

Multivariate analysis showed that lower RDW values were associated with higher rates of prosthesis‐free breathing at last follow‐up (OR, 0.54; 95% CI, 0.30–0.86; *p* = 0.017). Every 1 unit increase in RDW value showed a decrease in the odds of prosthesis‐free breathing by 0.54. Additionally, patients with tracheomalacia (OR, 0.19; CI, 0.04–0.79; *p* = 0.028) prior to intervention and patients with OSA (OR, 0.13; CI, 0.02–0.69; *p* = 0.024) were more likely to require a prosthesis for breathing at last follow‐up (Table 4). Patients with tracheomalacia comprised 48% of patients who required tracheostomy at last follow‐up and patients with OSA comprised 28%. RDW value was not associated with the need for further procedures following surgical intervention.

**TABLE 4:** Rate of prosthesis‐free breathing at last follow‐up.

Characteristic	Proportion (%)
Comorbidities	
Tracheomalacia	14/26 (54%)
Congenital cardiac conditions	31/38 (81%)
OSA	8/15 (53%)
Bronchopulmonary dysplasia	19/30 (63%)
Vocal cord paralysis	13/13 (100%)
RDW‐CV	
> 14%	33/48 (68%)
< 14%	38/47 (81%)
Age at surgery	
≥ 22 months	37/50 (74%)
< 22 months	36/48 (75%)

Abbreviation: OSA, obstructive sleep apnea.

## 4 Discussion

We hypothesized that serological markers and ratios may be used to stratify surgical outcomes in children with aerodigestive conditions. Using univariate and multivariate analyses, we found that higher RDW values were associated with poorer operative outcomes as measured by prosthesis‐free breathing at the end of the study period. In addition, we identified further serologic and disease‐associated risk factors for pediatric patients with aerodigestive conditions that correlated with increased healthcare consumption.

Though the exact number is uncertain, laryngotracheal stenosis has impacted a growing number of patients per year, given medical advancements [5]. Surgical management remains highly morbid with prolonged hospitalizations. Even with an aerodigestive workup informing the operative timing, at the population level, patients are experiencing reconstructive failures at unacceptably high rates. Inflammation appears to play significant roles in poor graft healing in LTR and restenosis in anastomotic operations [16, 17, 18, 19, 20, 21, 22]. Based on careful review of the literature regarding serological markers of inflammation available on routine preoperative studies, we sought to apply an analysis of inflammatory markers available on routine preoperative serology to help stratify inflammation in LTS surgical candidates.

Although the specific pathophysiology tying RDW to post‐operative outcomes has not been discovered, RDW is recognized as a non‐specific marker for inflammation, associated with all‐cause mortality [16, 17] in large population cohorts, as well as mortality from specific procedures [18, 19, 20, 21, 22]. This association is thought to be driven in part by oxidative stress, which accelerates erythrocyte senescence and clearance, and by an inflammatory milieu that favors the production of neutrophils and platelets [22, 23]. Proteomics analysis has found proteins such as IGFBP2 and others, involved in cellular senescence that could mediate the association between all‐cause mortality and RDW [24, 25]. A mechanistic link has also been proposed between elevated RDW and atherothrombotic events, the leading cause of postoperative complications [26].

While increased RDW is seen in conditions such as anemia due to vitamin deficiency or hemolysis, bone marrow dysfunction, and liver disease, its association with adverse outcomes persists even after accounting for anemia and hemoglobin levels [11, 15]. Previous otolaryngology studies have explored the association between RDW and outcomes of open airway reconstructive surgeries [27], laryngectomies [28], and its role as a prognostic indicator in upper aerodigestive tract cancers [29]. All studies suggested worse outcomes in patients with elevated RDW, likely due to the aforementioned inflammation and oxidative stress. Our results indicate that the RDW trends described in adults undergoing airway reconstructions and other aerodigestive interventions are likewise seen in pediatric patients. Lower RDW values in children undergoing airway interventions are associated with improved surgical outcomes.

The other serological markers of inflammation that we studied include MLR, SIRI, NLR, and PLR. These markers have gained increased interest in recent years as prognostic indicators for aerodigestive conditions. Elevated MLR and SIRI have been associated with the development of OSA [30, 31]. Elevated NLR has been associated with the development of asthma and its exacerbations in both adults [32] and children [33]. All of these markers have been studied in and associated with the prognosis of head and neck cancer [34, 35, 36, 37]. These markers are more directly correlated with the presence of an inflammatory process associated with RDW, and we saw this reflected in our results with all markers being associated with some aspect of complication or surgical recovery. In our cohort, preoperative serologies such as RDW were not used as determinants of whether to proceed with surgical intervention. Rather, we view them as markers of systemic inflammation that may carry prognostic value. While we do not currently alter our surgical decision‐making solely on the basis of an elevated RDW, these measures may provide valuable context in risk stratification. In particular, when planning major airway reconstructions, elevated inflammatory markers could signal an increased risk of failure attributable to untreated systemic inflammation. Addressing such inflammation preoperatively may improve the likelihood of successful outcomes. Ultimately, the precise way in which these markers influence endoscopic or reconstructive management will remain surgeon‐dependent, but they may help explain why certain patients remain inflamed or symptomatic after intervention.

Of the airway comorbidities that we studied, tracheomalacia and OSA required the most intense follow‐up and long‐term intervention. Tracheomalacia in the pediatric population is due to congenital or acquired impairment of tracheal cartilage integrity. Acquired tracheomalacia is caused by prolonged intubation while congenital tracheomalacia is associated with esophageal atresia and tracheoesophageal fistula [5]. Both are suggestive of extensive underlying comorbidities that could result in the association between tracheomalacia and the necessity for long‐term follow‐up and intervention. While OSA in the general pediatric population is most associated with tonsillar/adenoid hypertrophy, a patient population with LTS tends to have developmental and neurological comorbidities that render the anatomical causes of OSA more variable [38, 39]. A previous study has shown that patients with OSA that require tracheostomies usually have an associated syndromic diagnosis that results in upper airway obstruction, finding that the majority of children who undergo tracheostomy will remain dependent on it for at least 24 months [40]. This aligns with the extended dependence that we see in our study.

### 4.1 Limitations

Due to the constraints of the retrospective nature of our research project, many patients had an incomplete data set of potential serological studies. The different serological tests that were explored were described by different lab protocols with RDW being found in a CBC alone, while MLR, NLR, PLR, and SIRI are all calculated with a CBC with differential. In the present study, some patients only had CBC values. Fewer patients had a data set that included CBC with differential, and thus the patient populations supporting MLR, NLR, PLR, and SIRI are smaller.

The children that were presented for airway reconstruction often had diverse etiologies with complex histories of comorbidities. Due to this, it is difficult to parse out whether the length of hospitalization and follow‐up was entirely due to complications of the airway or if other factors were at play.

Classical lab tests for inflammation include erythrocyte sedimentation rate (ESR) and C reactive protein (CRP). In other conditions, CRP has yielded similar results to the serological tests that were studied. Although this is routinely ordered in adult populations, it is rarely used for children presenting for triple endoscopy or airway reconstruction. This meant that we were unable to compare our serologic tests to those that are considered gold standard measures for inflammation, to explore any correlation.

## 5 Conclusion

Our findings suggest that red blood cell distribution width (RDW) is a useful predictor of surgical outcomes in pediatric aerodigestive patients, with lower RDW‐CV values associated with higher rates of prosthesis‐free breathing. Additionally, inflammatory markers such as MLR, SIRI, NLR, and PLR were linked to aspects of surgical recovery, reinforcing their potential role in risk stratification. Given the complexity of aerodigestive conditions and their associated comorbidities, future studies should explore the integration of these biomarkers into clinical decision‐making to improve patient outcomes.

## Conflicts of Interest

The authors declare no conflicts of interest.

## Data Availability

The data that support the findings of this study are available from the corresponding author upon reasonable request.
